# Atoxyl: Suggested Use in Leuchæmia

**Published:** 1907-09-21

**Authors:** 


### Points in Treatment.

### ATOXYL: SUGGESTED USE IN LEUCHÆMIA.

Atoxyl owes its celebrity to its being the only drug which has proved at all efficient in the treatment of sleeping sickness. It has hitherto been used mainly in this disease, and has therefore not come prominently before medical practitioners at home. It is worth considering, however, whether or not it might be tried in certain diseases in which arsenic is our present mainstay, notably in the blood diseases other than chlorosis, especially leuchæmia.

### Arsenic and Aniline.

It has been known for some time that both arsenic and aniline, used independently, have some controlling effect in trypanosomiasis—that is to say, in that condition which is the prelude to sleeping sickness. Trypanosomes are protozoa of a particular kind, introduced into the human body by the bite of a fly, just as malaria parasites are; when the patient has trypanosomes in his blood only he has trypanosomiasis, and when these same trypanosomes have extended to the cerebro-spinal fluid he has sleeping sickness. Arsenic and aniline appear to act upon trypanosomes as quinine does upon malaria parasites, killing them when they are in certain stages of their development, but unfortunately not killing them when they are in other stages.

Seeing that each of these drugs acted in the same direction when given separately, attempts were made to combine the two, and the result has been the formation of *atoxyl*, a meta-anilide of arsenic. This has been found much more effective than either ingredient separately, and has two very great further advantages—namely, that whereas the benefit of arsenic alone is only temporary, and that whereas aniline cannot be pushed without producing nephritis, the effects of atoxyl are more lasting, and there is little or no fear of nephritis from it. Arsenic, moreover, when given as Fowler's solution for example, is liable to cause toxic symptoms—vomiting, peripheral neuritis, pigmentation of the skin—even in medicinal doses in certain people; atoxyl is at least twenty times less toxic, at any rate when given hypodermically. When given by the mouth it is more dangerous, because it decomposes and liberates arsenic in the gastric juice.

### What Atoxyl is.

The drug contains about one part of arsenic in two and a half. It is a powder, and as a powder it keeps good indefinitely, but to give it hypodermically it has to be made into a solution; the latter is readily decomposed by acids, alkalies, and light, and should therefore be made up fresh every few days. When the solution is kept it turns yellow or brownish, and arsenic is apparently set free; at any rate, the result is that the solution used after it has been kept a few days causes intense irritation, whereas the fresh solution is quite free from this objection.

### The Method of Administration.

The syringe used for giving the injection should be carefully sterilised by boiling; it should not be placed in any antiseptic, for such things as carbolic acid, for example, will decompose the atoxyl when the latter is drawn into the barrel. The atoxyl solution itself can be sterilised in an autoclave, or by simple boiling.

### Dosage.

There are various strengths used, varying from 5 per cent. to 20 per cent.; the solubility of atoxyl is such, however, that it remains dissolved in 20 per cent. solution only when the fluid is hot; the 5 per cent. solution, on the other hand, makes the volume of the injection rather large; a common strength is 7½ per cent.

The dosage in Belgium is not quite the same as that in London. In Brussels it is the custom, apparently, to inject that quantity of fluid which contains 3 grains of the atoxyl on the first day; to repeat the injection on the fourth or fifth day, increasing the amount to 3¾ grains; and to continue the injection every third, fourth, or fifth day, increasing each dose by ¾ grain until as much as 12 grains at a single dose is reached. Treatment in cases of trypanosomiasis is continued for two or three months, after which an interval of two months is given, the series being then repeated.

The practice in London is to begin with 1 grain, repeating the injections every second day, adding half a grain more each time until a dose of 3 grains is reached, this being then continued. A few cases have had as much as 8 grains twice a week.

### Intolerance.

The symptoms which indicate non-tolerance of the drug are similar to those of arsenic, but with less diarrhœa; violent headache and severe abdominal pain have been prominent in a few patients. Just as with other drugs, larger doses can be borne when the disease indicates its use than would be tolerated by a healthy person. The pulse-rate tends to become very slow.

### Atoxyl in Leuchæmia.

Atoxyl being a drug, therefore, which contains arsenic in a form which allows of far bigger doses being given than is possible with Fowler's solution, it seems possible that it may be of service in the treatment of leuchæmia, pernicious anæmia, and Hodgkin's disease. We are not aware of its having been tried in any of these; but seeing how hopeless the ultimate prognosis is in many of these cases upon ordinary lines of treatment, it would be well worth while to try atoxyl in some of them, using small doses such as a grain twice a week to begin with, and gradually increasing it.

